# International medical electives in selected African countries: a phenomenological study on host experience

**DOI:** 10.5116/ijme.5aed.682f

**Published:** 2018-05-23

**Authors:** Elspeth M. Fotheringham, Pippa Craig, Elina Tor

**Affiliations:** 1 School of Medicine, University of Notre Dame, Australia

**Keywords:** International medical elective, international medical student, host perspective, descriptive phenomenology, low- and middle-income countries.

## Abstract

**Objectives:**

To explore the host experience on international medical electives at a selection of hospitals in low- and middle-income countries in Africa. Outcomes of the study may inform and improve the preparation of global health curriculum, pre-elective training and debriefing for international medical electives.

**Methods:**

A descriptive phenomenological study was undertaken, involving semi-structured interviews with ten elective hosts at seven study sites in three African countries. Purposive convenience sampling augmented by snowballing was utilised to recruit study participants. The data were thematically analysed and interpreted with reflexivity to generate an accurate aggregate of the experience of participants in hosting international medical electives.

**Results:**

Six main themes emerged from the thematic analysis of interview data: international medical student contribution to host hospitals, host professional and personal fulfiment, barriers to student learning experience, international medical student preparedness, hope for reciprocity and barriers to cultural immersion and patient care.

**Conclusions:**

Study participants described the experience of hosting international medical elective students as overwhelmingly positive. However, they highlighted issues such as barriers to students’ learning experience and the lack of reciprocity between host and sending institutions as areas which could be addressed to optimize the experience for both hosts and international medical students. An understanding of the host experience provides stakeholders with a clearer idea of what is important in preparation, organisation and evaluation of the elective experience. This study provides the impetus for further research to examine the effectiveness of introducing appropriate pre-departure training and post-elective debriefing to students embarking on international medical electives.

## Introduction

There is a growing interest in global health education, as a result of globalisation.^1^ As interest in global health has grown, increasing numbers of medical students have chosen to participate in international medical electives (IMEs).^2^ The IME provides medical students with unique experiences to develop clinical skills and cultural competencies in diverse environments.^3^ Many students select elective sites in low-and-middle-income countries (LMICs) as it provides opportunities to develop clinical skills they would not acquire in their home country.^4^ There is much evidence from the literature to support the proposition that international electives are advantageous for students. Students report less dependence on technology; improved clinical, diagnostic, and communication skills; better knowledge of tropical diseases and immigrant health; and a better understanding of prevention, primary care and public health.^5^^, ^^6^

Despite the perceived benefits for students, very little research has been done to explore the benefits and challenges experienced by host institutions.^1^^,^^7^ In the Working Group on Ethics Guidelines for Global Health Training,^1^ potential challenges were identified. IMEs may create substantial burdens to the host in a resource-poor setting, may have a negative impact on patients, the community and local trainees, and can create unbalanced relationships amongst institutions and trainees which result in difficulties with ongoing sustainability and resource utilization.^1^

Recent small qualitative studies have provided some understanding of the host perspective. In one such study,^8^ participants expressed both positive and negative experiences associated with visiting health professionals. Participants articulated the importance of visiting health professionals attending effective pre-departure training with information about the cultural and environmental context of the host institution; the value in forming long-term partnerships that are mutually beneficial to both host and visitor; and the importance of the visiting health professional demonstrating “cultural humility”^9^ by showing respect, humility and a desire to learn from the community which hosts them. A small questionnaire-based study,^10^ identified the need to minimize the harm that could result from IMEs, including the impact on limited resources and patient care. Another highlighted the importance of reciprocity,^11^ where partnerships between sending institutions and host hospitals could be mutually beneficial.

Some medical schools have responded with improved global health training by providing appropriate pre-elective training and post-elective debriefing for students as well as developing partnerships and collaboration with host institutions.^12^^,^^13^ However, much work still needs to be done with further research needed to ‘address the educational objectives of IMEs and the impact these activities have on trainees and host communities’.^2^

In the Australian context, medical students undertake electives at some stage during the latter part of their training: electives are a compulsory component of all medical curricula. Approximately 60% of Australian students undertake IMEs in LMICs.^14^ Pre-departure training, and post-elective debriefing is not currently offered to all students, and when it is, it is not compulsory. The absence of such processes was seen as sub-optimal in one study,^14^ which recommended scaling up of both pre-departure training and post-elective debriefing across Australian medical schools.

This study aims to gain insight into the experience of individuals who host medical students to inform the development of a suite of appropriate processes including pre-departure training and post-elective debriefing.

Insight was sought into the following two questions:

1.     What is the experience of individuals directly involved in hosting international medical students for medical electives in a selection of hospitals in LMICs in Africa?

2.     How can this experience be optimised for both host institutions and international medical students?

## Methods

### Study design

We conducted a phenomenological study to describe the experience of individuals hosting international medical students (IMS) at a selection of hospitals in LMICs^15^ in Africa. The philosophy underpinning the research was descriptive phenomenology as we seek to explore the ‘lived experiences’ of the participants.^16^ A constructivist and interpretivist lens allowed us to relate the experience of hosts to existing knowledge of IMEs and deepen our understanding of the host perspective.

### Participants

This study received approval from the University of Notre Dame Australia research ethics committee. Additional ethics approval was obtained from two academic institutions, and reciprocal approval was obtained from a third institution in the hosting LMICs.  Hospital permission was obtained from hosting hospitals and informed consent collected from host participants. 

The participants recruited were all involved with IMS but their involvement varied, thus providing different perspectives and enriching the data.^17^ Thirty-two host participants were approached and informed of the study by email; of these, ten agreed to be interviewed subject to ethics approval being obtained from their institutions. The participants comprised nine doctors whose roles varied, but included supervision, administration, orientation, mentoring and pastoral care of elective students and one elective coordinator who was responsible for the organisation, administration, allocation of students and pastoral care. There were six males and four female participants from three African countries.

Participants were recruited using purposive convenience sampling, leveraging links between the principal investigator and known providers of electives in the region. An additional participant was recruited through snowball sampling. Delays in obtaining local ethics approval was a rate-limiting step to both recruitment and collection of data. Saturation of data was reached after nine interviews as no new themes were identified.

### Data collection

Data was collected through one-on-one, semi-structured interviews using an interview guide (Box 1). The questions were sufficiently broad and open-ended, so the interviewee would have the opportunity to ‘express his or her viewpoint extensively’ in keeping with the approach to the phenomenological interview as described by Bevan.^17^

**Box 1:** Interview guide: sample questions

· What has been your experience of hosting IMS, in particular, Australian medical students?
· Do IMS affect the way in which you are able to deliver health care, if so how?
· What is the attitude of local students to IMS?
· What is the attitude of patients/local community to IMS?
· How can this experience be optimized to maximize benefits and limit harm?
· What suggestions do you have for health educators in Australia to better prepare students for the experience at your institution?

Seven interviews were conducted face-to-face in the host country during March 2017. Three interviews were conducted via video conference from a venue convenient for both researcher and participant on account of geographical distance. Interviews lasted 25-30 minutes. The principal investigator conducted, and audio recorded each interview.

### Procedure and data analysis

Measures to ensure rigour and trustworthiness of data and results included firstly recruitment of participants with different levels of involvement in hosting IMS.  Audio taping of interviews also allowed the researcher, EF, to listen to them multiple times, which was important for thematic analysis. Participants’ consent was obtained to include relevant quotes in the presentation of findings from the study.

Interviews were anonymized during transcription. In descriptive phenomenology, as described by Husserl,^18^ the researcher has the potential to bias the research through personal interpretations. Bracketing involves the investigator ‘putting aside’ their preconceptions and perspectives.^18^ Researcher EF is originally from the southern African region and is familiar with the context, culture and health care system of that region. EF has attempted to minimize bias by ‘bracketing’ this knowledge during data collection and analysis.

EF transcribed the recorded interviews, coded and analysed using thematic analysis as described by Braun and Clarke.^19^ NVIVO computer software was used to code data and identify themes and subthemes (Version 11, QSR International).  The data included four hundred and eighty coded quotations, from which over forty sub-themes were  initially generated. Themes were regrouped and collated. EF and PC met to discuss the coding structure and emerging analytic themes.

### Setting

Participants were from seven different elective sites located in three African countries. There was one site in Uganda, five in South Africa and one in the Kingdom of Swaziland (see relevant information regarding site and number of interviewed participants in Table 1).

**Table 1:** Participant distribution across different type of host sites

Type of Host Site	Number of Hospitals in each type of host site	Geographical Location for each type of host site	Number of participants from each type of host site
Hospital: Church/Government partnership	2	Rural	3
Government Hospital	1	Rural	1
Government Hospital	1	Urban	1
Academic/Teaching Hospital: University-affiliated	3	Urban	5

In addition, participants also provided background information about study context including the country of origin of IME students, length of elective, favoured disciplines, supervisor experience and elective intake (Table 2). 

**Table 2:** Summary of international medical elective programs

Student country of origin	UK, Germany, Netherlands, Scandinavia, Australia, Belgium, France, USA
Length of elective	4 to 16 weeks (most common is 4 weeks)
Favoured disciplines	Trauma, Surgery, Obstetrics/Gynaecology, Emergency Medicine, Paediatrics
Supervisor experience	3 to 37 years
Elective intake	7 to 300/year per institution

## Results

Six main themes emerged during data analysis. They were: IMS contribution to host hospitals; host professional and personal fulfilment; barriers to IMS learning experience; IMS preparedness; desirability of reciprocity, and; barriers to cultural immersion and patient care (Table 3).

**Table 3:** Summary of main themes and sub-themes emerging from data

Main Theme	Sub Theme
IMS contribution to host hospitals	Financial	Human resources	The mutually beneficial learning experience
Professional and personal fulfilment for host	Not applicable
Barriers to IMS learning experience	Clarifying learning objectives and level of experience	Variability in supervision
IMS preparedness	Familiarity with local population health profile	Cultural awareness and humility	Pre-elective preparation	Student safety
Hope for reciprocity	Not applicable
Barriers to cultural immersion and patient care	Not applicable

These themes will now be explored in detail illustrated by relevant quotes from participant interviewed. Interview numbers identify different participants.

### IMS Contribution to Host Hospitals

#### Financial

Participants felt that hosting medical students benefited the hospital financially as host hospitals charged IMS an administrative fee. Teaching hospitals affiliated to universities charged an additional international student registration fee, which was paid to the university administration. Most hosts felt that the hospital fee was valuable, as it was used to cover tuition, equipment and consumables (e.g. gloves) used by students. Two participants described additional benefits:

“the hospital gets a ready source of income which is actually valuable in a state system where there’s no money for projects, so it was often used as conference money or for rehabilitation of grounds, so it created a fund that the hospital could use for projects that benefited the hospital.” No 1

“we try to make sure that half of it goes towards subsidising treatment for patients who can’t afford to pay.” No 2

Additional funds were also received from student-run organisations.

“some of them come back again years later as volunteers; quite frequently they become engaged in our donor activities.” No 9

These funds were often used for specific projects and reflected an ongoing relationship between past elective students and their hosts.

#### Human resources

Participants felt that IMS made a valuable human resource contribution to hospitals. Students provided practical assistance in wards, theatres and emergency departments:

“extensive use is made of students assisting in surgical procedures after hours.” No 1

Their youth and energy boosted the morale of permanent staff who often worked in under-resourced, poorly equipped conditions. IMS provided a different perspective of medicine; they questioned standards of practice which they observed were not evidence-based and highlighted the importance of research in medicine. These sentiments were expressed by one participant:

“I think, the biggest contribution (elective) students make in terms of medical education is helping to demonstrate a different way of learning and an openness to things like research.” No 2

Participants reported that IMS contributed to patient care and due to the huge burden of need, did not feel competition between IMS and local trainees was a significant problem. One participant explained that for similar reasons patients were happy to be treated by students and in some instances, were unable to discriminate between doctor and student:

“If you (patient) are getting a good service from your health care provider like you’re getting an interested, detailed history and a proper examination, they’re going to be happy, so they appreciate the time spent.” No 3

#### Mutually benefitting learning experience

The opportunity for shared learning was mentioned by most participants when IMS worked alongside local doctors and trainees in teams or within specialist units. Most participants felt that despite differences in clinical skills, knowledge and level of competence, local trainees and IMS gained from the shared learning experience which in some instances led to a greater awareness of the strengths and weaknesses of their medical curriculum and an appreciation of different ways of learning:

“I think we (host) are far less didactic and it’s kind of watch one, teach one, do one kind of thing, and they (elective student) have far more structured learning, so it’s always nice to compare.  I’m not saying that one’s better than the other, it’s just we learn from each other.  So, it’s always been very positive.” No 6

IMS contributed to existing research projects and the development of site-specific treatment protocols and guidelines appropriate to the host context.

Some participants felt early debriefing during the first week of the elective and regular tutorials would enhance learning for students unfamiliar with the scope of pathology and the severity of the disease. Participants felt a formal “exit” interview at the end of the elective period would provide valuable learning for themselves as they would get feedback from the student about the elective experience and what improvements could be made.

### Host Professional and Personal Fulfilment

Host participants described personal satisfaction and professional fulfilment as motivators to hosting students.

“We see our role here as planting seeds for international health, for rural health, for the rights of people who are disadvantaged.” No 9

They felt that by hosting students, they could hand the baton on to a future generation of doctors and pay back something to the profession. Some felt paternalistic towards the students and had a mentoring role. Others felt it was their duty to introduce students to a different cultural experience which could stimulate an interest in global and rural health and promote a culture of global citizenship.

### Barriers to IMS learning experience during IME

#### Clarifying learning objectives and level of experience

Most participants expressed a desire to know more about the aim and purpose of the elective from the student and sending institution. This included information about the educational objectives and student learning goals:“All that we’re aware of is the lack of practical experience, so we focus on that, and they usually want to do procedures. So, if we were to know something more about what they actually are expected to do and what they need to pass their exams, maybe that would help us in terms of direction.” No 1

Improved understanding of the level of student experience was felt to be important and could guide student orientation, placement and level of supervision:

“I think it’s also useful to know what their level of exposure and experience is and what they’re able to do, what they’re not able to do.” No 6

Participants reported that assessment of students’ performance during the elective period was not aligned to learning objectives and the type of assessment expected by sending institutions from the hosts was variable.

#### Variability in supervision

Due to large student numbers in some hospitals, participants felt that the level of student supervision varied and depended on individual department heads and supervisors.

“Some doctors find the students a bit of a handbrake and especially if they’re only here for a month it’s hard to train, and they’ve got to rotate through your department every week.  It’s hard to invest in them because they’re gone next week, and so some doctors just don’t invest in them.” No 9

Hosts expressed reservations that if inadequately supervised, students may exceed boundaries of competence, performing procedures that they would not be permitted to do in their home countries and not necessarily learning the right way of doing things. They acknowledged that not all clinicians in their hospitals were interested in teaching elective students as there was no real incentive in doing so, and they had other clinical or teaching responsibilities:

### IMS Preparedness

#### Familiarity with local population health profile

Participants felt IMS should be familiar with the geographical burden of disease and that site-specific preparation was important. Some host participants provided locally relevant guidelines to assist students in their preparation and suggested students use a log book to record developing competencies:

“I (host) can send them electronic guidelines and stuff they can have on their phones so they’ve got something available all the time, because it’s no good having your hypertension guidelines for your country if those drugs aren’t available.” No 3

#### Cultural awareness and cultural humility

Some participants felt as students were guests in a foreign country, they should behave accordingly:

“Firstly, that they are visitors in a host country and they need to respect the customs and the culture to which they are coming, which is very different to their home culture.” No 7

Others felt that pre-elective preparation should include cultural awareness training so that students would be aware of their own cultural bias and be mindful of the importance of ‘cultural humility’:

“I think perhaps the preparation needed would be to help them (elective student) understand how they see their practice of medicine as having a cultural bias to it.” No 9

Some students struggled to make the shift to working in a poorly resourced environment so different to their own. Standards of care were different from their own countries and this was difficult to accept, particularly when faced with patient suffering and death. Participants felt cultural training would provide students with a better understanding of the host context and how health care systems differ. Some participants felt this training would help students to be more flexible and adaptable as doctors in the future:

“So, they almost need to be prepared for what to do in a situation where you’re seeing things not managed optimally as they should be for whatever reason.” No 2

“we often have to make do with what we have and do what we can with the resources we have immediately available, so it’s getting that expectation that the CT scans are difficult to get and they’re 2 hours away.” No 3

Most participants felt students needed to understand the importance of relationship in African culture. They felt it was useful for students to know how to interact with local staff particularly nursing staff:

“You had to greet (nurses) and ask how they are, have a little nice chat that is actually human decency which we have lost in the West.  So basically, it’s human relationships come first before what has to happen.” No 3

Understanding the professional role of local nurses was considered important by participants, as students expected them to have the same professional role as nurses in their own countries.

#### Pre-elective preparation

Early application for elective placements was encouraged by host participants who felt students underestimated the time

and administrative effort required by the host to organise electives. One participant commented it would be useful to screen students for their suitability for an elective as some students found their particular electives challenging. Some participants observed that older mature students seemed to have higher resilience and coped better:

“Mature students do well because they can be resilient and think, okay, this isn’t great, but I’ll have to make a plan.” No 3

#### Student safety

Participants highlighted that student safety was one of the main concerns for them when hosting students on electives:

“I think one of the things that living and working in Africa I would often be concerned about is their personal safety because they weren’t aware of the dangers.” No 1

Identified dangers included exposure to crime and personal injury, road accidents and risk of exposure to infectious diseases particularly as certain host countries had high rates of HIV and TB. Participants felt it was important to include aspects of personal safety training during pre-elective training and orientation.

### Desirability of reciprocity

Most participants expressed a desire for greater reciprocity between sending institutions and host hospitals as they felt the relationship was generally one-sided. They expressed a desire to look at partnerships where students or registrars from both host and sending institutions could be part of an exchange programme to experience medicine in a different culture:

“The one area that I would like to explore is students can come and learn from us, but could we send our fellows training in sub-specialties to them because they would be more developed.” No 5

One participant felt that some countries where low patient numbers limited the level of clinical experience and training, students were outsourced to developing countries to improve their practical skills:

“From the way I understood it, certainly in Germany, is that they trained three times as many doctors as they actually have jobs for and they farm them out at every stage.” No 1

### Barriers to cultural immersion and patient care

One of the concerns of some participants was the lack of cultural immersion as IMS had limited contact with the host community outside the hospital environment, something a participant felt was regrettable as it diminished the understanding of the patients’ cultural context:

“I try to give them (IMS) an experience of meeting African people, outside of the hospital, where they can socialise with people of the community and realise that there’s a life behind their patients.” No 5

Some participants observed that IMS from the same country seemed to stick together as a group and although that provided companionship and a shared experience in a foreign country, large groups meant that students were less integrated and, in some instances, less accountable:

“They might all duck off at lunchtime, particularly if a big group of five all come at once.” No 3

Some felt that language barriers and cultural differences in expressing emotions resulted in miscommunication and misunderstanding of local patients by students:

“Sometimes they (students) got the impression the patient does not care, and you go, no, it’s not that they don’t care, it’s just that they’re not expressing the emotion as you would have expected them to express the emotion.” No 8

Participants felt longer electives would be mutually beneficial as they thought it took students at least two weeks to become culturally adapted and adjusted to host organisation structure before they could contribute clinically:

“It’s most disappointing when students are all of a sudden oriented and comfortable and productive in their new environment and then that’s usually around about the one-month mark and then they go.” No 9

## Discussion

There have been many studies that have documented the benefits and challenges of IMEs for IMS. However, few studies have considered the elective experience from the host perspective. In this study, we sought to describe the experience of individuals who host IMS during IMEs. These findings provide useful insight into understanding the host experience and allow us to consider how this experience can be optimised for both host institutions and students.

Participants in this study expressed that IMEs provide a unique opportunity for experiential learning which can be very enriching for IMS, as well as for local staff and students. Local staff and students benefit from being exposed to a different approach to medicine, including openness to research and the value of continuing medical education. This finding is consistent with a previous study, where host trainees learnt alongside international trainees in a Kenyan setting,^12^ and is in contrast to potential challenges suggested by the WEIGHT guidelines.^1^ Important learning opportunities for IMS identified by the hosts and in other studies^5^^,^^7^ include skills acquisition, increased awareness of global health and the importance of the elective being as much a cultural experience as medical experience.

However, participants felt the learning experience for both IMS and host could be further improved. Participants in our study highlighted the variability in supervision for IMS, this is consistent with what has been reported by Kumwenda et al.^11^ Regrettably participants felt that not all clinicians in their hospitals were interested in teaching IMS, and there were few formal teaching sessions or other types of support provided for IMS who were often just expected to fit in. Formal teaching sessions for IMS and early debriefing by the host was proposed by participants who felt these initiatives would enhance student learning and provide IMS with support during the early intra-elective period. Participants suggested IMS to use a logbook to record their learning and development. The inclusion of an ‘exit’ interview with opportunity for feedback would provide the host with valuable information to improve the learning experience for future IMS.

In the same vein, participants emphasised the importance of pre-elective preparation. Clear learning objectives and an improved understanding of IMS level of experience were felt to be important. The lack of clarity concerning the educational objectives of electives has been previously highlighted by Cherniak and colleagues,^2^ who emphasised the need to develop core competencies for global health and specific educational objectives for electives. If hosts had a clearer idea of elective objectives and students’ learning goals, they would be able to provide direction, and suitable placement matched to student year of study, skill set and level of competence. Understanding the level of experience would reduce the likelihood of IMS exceeding boundaries of competence in clinical practice during their IME.^12^ Educational objectives and goals could also be set in collaboration with the student and the host as suggested in other studies.^8^^,^^9^

Participants felt that IMS preparation should include not just an understanding of the geographical burden of disease, but also an awareness of their own cultural bias which would lead to a greater understanding of how medicine is practised in the host country. Awareness of their own cultural bias should be included in IMS preparation. This practice is in keeping with the description of cultural humility,^9^ ‘the concept of respect and curiosity toward cultures other than one’s own’, which is described as one of the most important global health competencies.^9^ Other studies have indicated that respecting the cultural environment of the host is important for visiting health professionals.^8^ Some host participants currently provide locally relevant guidelines. However appropriate pre-departure training could better equip students to work in resource-poor settings with limited supervision.^12^

Findings from this study indicate that site-specific cultural competency training would also be useful. This deficit in preparation was also highlighted in a previous study,^11^ and could include language and site-specific cultural training as well as highlighting safety issues when working and travelling in the host country. Understanding the work, culture and role of other health professionals, in particular, the role of nurses would be helpful to prevent miscommunication and misunderstanding.

Another recommendation relating to student preparation and elective organisation considered to be important by the hosts in this study also identified elsewhere,^4^^,^^11^ would be to increase the length of the elective period to benefit both the student and the host mutually. Hosts could be more involved in student selection or screening to assess the suitability of the student to their local context. This finding is supported by other studies which consider the host perspective.^8^^,^^11^ Some participants in this and one other study^11^ felt that older, more mature students seemed to have higher resilience. This has implications in Australia, where almost half of the medical schools are graduate entry with older students; we know that more than half of Australian medical students undertaking IMEs do so in developing countries.^14^

Participants felt that hosting IMS provided a ready source of income for hospitals, useful for projects, research and supplementing patient care. Additional funds were also received from student-run NGO’s. Some IMS came back as volunteers, establishing an ongoing relationship which continued to benefit the hospital. This trend is encouraging as it shows that IMEs do achieve the overarching goals for global health by helping to develop IMS global citizenship.^5^ The financial benefits of hosting medical students have been identified in a previous study.^11^ This finding is in contrast to potential challenges cited by Crump^1^ who suggested students could be a substantial burden on the host community.

Participants acknowledged that IMS made a valuable human resource contribution to hospitals working alongside local doctors and trainees. Most participants felt that local students and IMS gained from the shared learning experience. Participants described a collegial relationship and, due to large patient numbers, did not feel competition between local and IMS students was a significant problem. Patients themselves were happy to be seen by IMS due to the huge burden of need in LMICs; those from an underprivileged background are unable to discriminate between a doctor and student. This perception does raise ethical concerns previously raised by Crump^1^ as these patients are vulnerable and disadvantaged; but due to the burden of need in the local context, participants did not feel this was a problem.

Few participants described any formal partnerships or links with sending institutions. They expressed a desire for such alliances, where local students or registrars could be part of an exchange programme to experience the practice of medicine in a different culture; this idea of reciprocity has been highlighted in the previous literature.^1^ Bi-directional partnerships between host and sending institutions, although desired by the host, were considered as being unlikely to develop due to many barriers for students from LMICs travelling abroad and no clear commitment from sending institutions. Successful examples of bidirectional partnerships exist,^12^ host and sending institutions could use these as models to develop similar relationships.

Significant barriers to cultural immersion observed by participants included their observations that larger student groups travelling together were less likely to mix with locals and that a lack of cultural understanding and language difficulties could be a significant barrier to patient communication. Participants felt that IMS needed to understand that communication and developing relationships were important in the African context. The concept of relationship-building is supported in the literature.^8^ This is applicable in the hospital environment where there was an expectation that IMS would be aware of these cultural expectations and communicate with local hospital staff appropriately.

We acknowledge that our study has limitations. This was a small exploratory study with information obtained from elective sites limited to a specific geographical region on one continent. The small scale of the research may potentially restrict the transferability of study findings. However, while some results are obviously site-specific, some themes are sufficiently broad to be considered more generally. We acknowledge that the elective providers interviewed are likely to be self-selected and that disinterested, or less motivated participants could have been excluded. EF’s connection to the region is both a strength and weakness. This connection provided links to known elective providers and knowledge of the region, context, culture and health care system. However, this prior knowledge could potentially introduce bias during data collection and analysis and is considered a possible limitation, although EF consciously attempted to address this bias through ‘bracketing’.^18^

## Conclusions

Study participants described the experience of hosting international medical elective students as overwhelmingly positive. They have, at the same time, highlighted issues such as barriers to students’ learning experience and the lack of reciprocity between host and sending institutions as areas which could be addressed to optimize the experience for both the host and IMS. These findings contribute to a better understanding of the host experience, which will provide all stakeholders involved in organising IMEs with a clearer idea of what is important in the preparation, organisation, participation and evaluation of the elective experience. This exploratory qualitative study could provide a platform for further quantitative research examining the effectiveness of introducing pre-elective training and post-elective debriefing for students embarking on IMEs. One area worthy of future research on IMEs is to look at how and why students select specific sites to do their IMEs and what the requirements are for a worthwhile elective experience considering both student needs and host factors. This may enable sending institutions to develop educational objectives with the joint collaboration of students and their hosts.

### Acknowledgements 

The authors wish to thank all the hospital staff at elective host sites who participated in the interviews.  The anonymity of these persons is guaranteed.

### Conflict of Interest

The authors declare that they have no conflict of interest.
